# Microbial Food Safety Assessment of Organic Food and Feed: Notifications in the EU RASFF during 2020–2022. A Systematic Review

**DOI:** 10.1155/2023/6615992

**Published:** 2023-11-25

**Authors:** C. Rodriguez, H. Mith, B. Taminiau, N. Korsak, E. Garcia-Fuentes, G. Daube

**Affiliations:** ^1^Instituto de Investigación Biomédica de Málaga y Plataforma en Nanomedicina-IBIMA Plataforma BIONAND, 29590, Málaga, Spain; ^2^UGC de Aparato Digestivo, Hospital Universitario Virgen de la Victoria, 29010, Málaga, Spain; ^3^Fundamental and Applied Research for Animals and Health (FARAH), Department of Food Microbiology, Faculty of Veterinary Medicine, University of Liège, Liège 4000, Belgium; ^4^Research and Innovation Center, Institute of Technology of Cambodia, Russian Federation Boulevard, P.O. Box 86 12156, Phom Penh, Cambodia

## Abstract

The presence of pathogenic bacteria in organic feed and food is a topic of current subject of study due to concerns that certain organic practices may heighten the risk of contamination and contribute to the spread of foodborne pathogens. The primary objective of this research is to analyze the main microbial contaminants exclusively associated with organic products, as reported in the literature. The search and selection of suitable studies were conducted following the Preferred Reporting Items for Systematic Reviews and Meta-Analysis guidelines. Databases consulted included Web of Science Core Collection, Medline (PudMed) database, and UE Rapid Alert System for Food and Feed (RASFF) database. Using the EU RASFF System, we have summarized the notifications in relation to these products during the last 3 years in Europe. Eligibility criteria were studies published in English between 2000 and 2022. All authors performed critical appraisal and independent data extraction. Analysis of RASFF notifications related to organic products over the period from January 2020 to October 2022 revealed that 61.7% of notifications were related to food, while only 38.2% were related to feed. *Salmonella* emerged as the predominant pathogen reported in both organic food and feed. Notably, only one food outbreak linked to *Salmonella* Enteritidis in eggs was reported during the study period. Among food commodities, seeds were the most frequently affected, with the highest percentage of food products with alerts originating from India. Regarding antimicrobial resistance, a noteworthy trend was observed, with a decrease in multidrug-resistant strains favoring organic production compared to conventional methods. In conclusion, this research aimed to investigate the presence of pathogenic bacteria in organic feed and food, considering the potential risk factors associated with organic practices and their implications for food safety.

## 1. Introduction

Organic food has experienced significant growth in recent years due to increased consumer demand, heightened concern for environmental health, and a sustained commitment to sustainable development within the framework of the green economy [1, 2]. Organic farming practices appear to contribute to optimal health status and decrease the risk of developing chronic diseases, perhaps due to the lower content of heavy metals, synthetic fertilizers, and pesticides [3].

Antibiotic use is less intensive in organic production, which could have a key impact on the circulation of antibiotic-resistance strains in both human and animal populations. A previous study showed how tetracycline and fluoroquinolone-resistant rates were lower in *Campylobacter jejuni* (*C. jejuni*) isolates from organic Turkey meat compared with conventional meat [4]. In the United States, a multicenter study described a lower prevalence of multidrug-resistant organisms (*Salmonella*, *Campylobacter*, *Enterococcus*, *Escherichia coli*) in organic meats than in the same conventional products [5]. However, other studies also reported no statistically significant differences between organic and conventional products in terms of circulation of antibiotic resistance genes. This was the case for *Staphylococcus* spp. in fresh cheese in Brazil [6], *E. coli* from chicken products in Korea [7], and tetracycline and sulfadiazine-resistant bacteria in organic lettuce [8].

In this context, organic food may have a significant load of enteric pathogenic bacteria, yeasts, viruses, toxins, or metalloids. Practices such as the use of natural animal manure [9] or water sources [10] may increase the risk of contamination of fresh organic produce and contribute to the spread of foodborne pathogens. For example, the lack of antimicrobial use in organic production was associated with a higher prevalence of *Salmonella* in fresh produce [11, 12]. Higher percentages of *Campylobacter* have been detected on free-range chicken and pig farms when compared to conventional housing [13–15]. In contrast, the presence of *E. coli* O157:H7 appears to vary as a function of animal feed, which was reduced when high proportions of roughage were added to the diet of organic livestock [16]. Gonzalez et al. [17] demonstrated that organic farming is not free from environmental contaminants, in particular polychlorinated biphenyls, polychlorinated dibenzo-p-dioxins, and dibenzofurans, among others, with a similar presence in organic and conventional foods. Similarly, the extensive use of wastewater in organic farming led to the presence and accumulation of heavy metals in soil and plants [18].

In this study, a systematic review was conducted using the Preferred Reporting Items for Systematic Reviews and Meta-Analysis (PRISMA) guidelines to ensure a comprehensive and transparent approach. PRISMA provided a well-structured framework for conducting systematic reviews in order to identify, assess, and synthesize relevant studies effectively. First, in this review, the main microbial hazards associated exclusively with organic products were analyzed as reported in the literature. Second, using the EU Rapid Alert System, we identified the main hazards found in organic feed and food, and we summarized the notifications in relation to these products over the last 3 years in Europe.

## 2. Literature and Data Research

**Figure 1:**
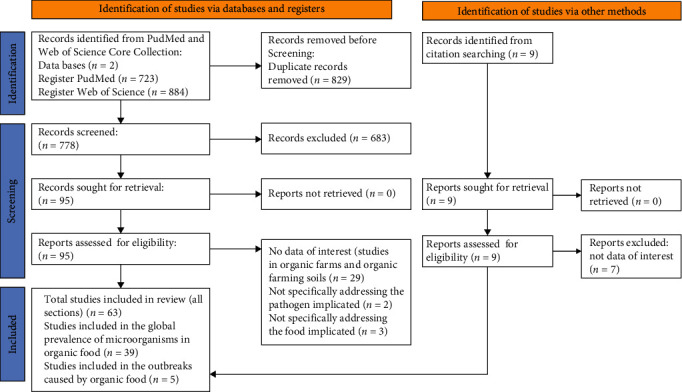
PRISMA 2020 flow diagram for new systematic reviews, which included searches of databases, registers, and sources.

A literature search was conducted in Medline (PubMed) and Web of Science Core collection. The search and selection of suitable studies followed the PRISMA [19] and according to the PRISMA 2020 checklist. A PRISMA 2020 flow diagram for new systematic reviews, which involved searches of databases, registers, and other sources, is included in Figure 1. The articles included in this review were selected from the Web of Science Core Collection and Medline (PubMed) databases. Articles referenced in the search results were also analyzed. In order to obtain as much data as possible, articles considered for inclusion were any review, full-research article, or short communication published in English between January 1, 2000 and December 31, 2022. A shorter period significantly reduced the available data in organic food. The following exclusion criteria were applied: studies involving outbreaks of bacteria and virus in nonorganic food or feed and studies conducted in a mixture of organic and nonorganic foods. Studies reporting mycotoxin contamination were also excluded.

Index search terms included (“organic food” OR “organic feed,” OR “organic produce,” OR “bio products,” OR “outbreaks,” OR “pathogens,” AND (“bacteria,” OR “virus”), AND (“*Campylobacter*,” OR “*Salmonella*,” OR “*E. coli*,” OR “*Listeria*,” OR “*Staphylococcus*,” OR “*Enterococcus*,” OR “*Clostridium*”)). This formula was used for PubMed (MeSH) and Web of Science Core Collection searches. Two authors independently reviewed the pool of articles based on predetermined inclusion, and they selected the articles and extracted data. A final consensus was reached with three additional arbitrators. This method enhances the reliability and objectivity of the article selection and data extraction, reducing the impact of individual biases and increasing the overall quality of the review. The outcomes sought in this study included investigating the distribution and prevalence of microbial pathogens in organic food and feed products. This involves studies analyzing epidemiological data, conducting surveillance studies, and reporting of foodborne outbreaks. The data search was carried out taking into account a number of variables, including the characteristics of the food or feed (type, origin, production practices) and the identification of microbial pathogens.

The Rapid Alert System for Food and Feed (RASFF) is a European database that collects information on identified risks to human and animal health in the food and feed chain and the measures taken. The aim of this system is, therefore, to exchange information and to help each national competent authority to harmonize the general principles and requirements of food law based on Regulation EC/178/2002. For microbiological criteria, Commission Regulation (EC) No. 2073/2005, applicable from January 2006, lays down food safety criteria for relevant foodborne bacteria, their toxins, and metabolites. The EU Rapid Alert System database provides information on food and feed safety alerts, notifications, and recalls for the past 2 years. The historical data older than 2021 was available through the EU open data portal (Distributions, RASFF notification pre-2021). The search conducted on the RASFF Portal involved the use of the terms “organic products,” AND “bio products,” AND “bacteria,” AND “viruses.” The index search criteria included “Countries: any;” “Product category: any,” “Type: any;” “Subject: organic and bio;” “Risk and hazard category: pathogenic microorganisms,” bio-contaminants and not determined/others,” in order to ensure that no information was lost by classification. This search included the selection of the following fields: “product category,” “pathogen,” “type of product.” “country (notifying, origin, distribution),” “type of notification,” “measures,” “human cases,” “risk decision,” “year,” and “EU RASFF reference.” The search was limited to notifications from January 1, 2020 to October 28, 2022. Mycotoxins, biotoxins, and nonpathogenic microorganisms (such as mold infestation) were eliminated as they did not fit the subject of the article.

## 3. Fecal Indicator Bacteria and Microbial Pathogens in Organic Products

Organic production systems are now a popular alternative to classical intensive farming practices, mainly associated with improved animal welfare standards and a higher quality end product. However, these practices were currently associated with a risk of transfer of bacterial pathogens to livestock and other food safety problems [20].  Table 1 presents data obtained from studies comparing the prevalence and presence of antibiotic-resistant strains in organically produced food compared to conventionally produced food. The presence of fecal indicator bacteria, including total and fecal coliforms, *Escherichia coli* (*E. coli*), and *Enterococcus*, among others, has been classically associated with fecal contamination when detected in water, foods, environment, or even soils [31]. Previous studies have addressed the presence of these hazards in outdoor and organic farming systems in relation to human health and focused mainly to livestock and food production environments [16, 18, 20], while only a few described outbreaks in organically produced products.

**Figure 2:**
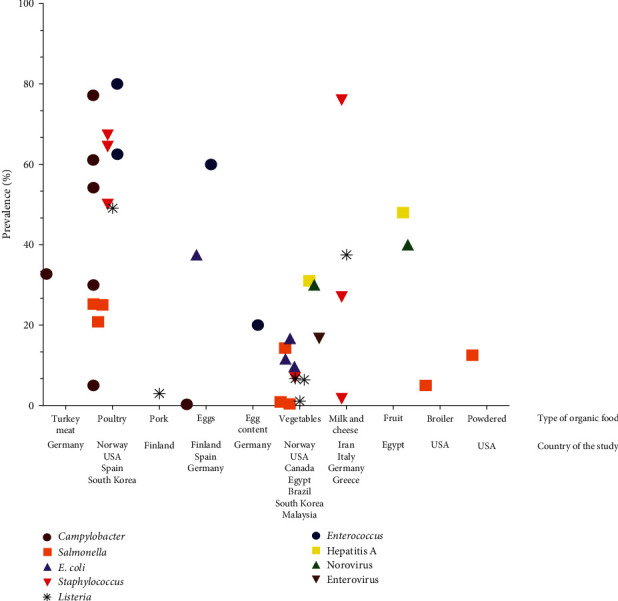
Global prevalence of fecal indicator bacteria and microbial pathogens in organic products. Data were taken from 39 studies in 13 countries worldwide.

**Figure 3:**
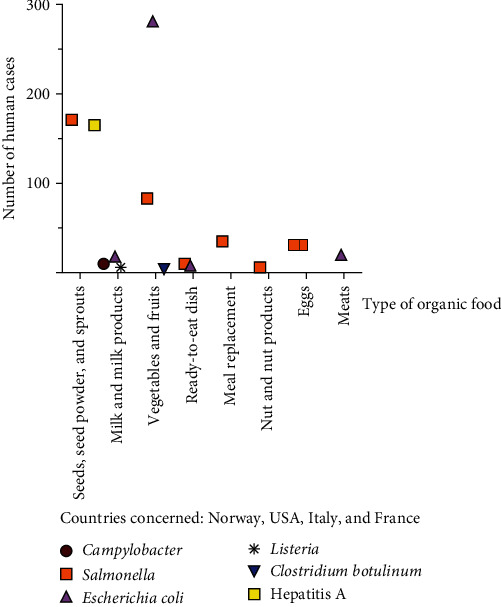
Reported outbreaks caused by organic foods in Europe and in the United States in the scientific literature. Categories: milk and milk products (raw milk, cream, cheese); eggs; vegetables and fruits (vegetable-based soup, tomatoes, lettuce, spinach, pasteurized carrot juice); seeds, seed powder, and sprouts (chia seed, alfalfa sprouts, fenugreek sprouts, pomegranate seed); meat (fermented meat sausage); ready-to-eat dish (sweet potato dish); meal replacement (organic powdered smoothie); nut and nut products (nut butter).

Regarding antimicrobial resistances, *Enterococcus* species easily acquire antibiotic resistance through the horizontal transfer of mobile genetic elements, and this process is currently used to study the spread of bacterial multiresistance in conventional and organic meat products [24].  Figure 2 illustrates the global prevalence of fecal indicator bacteria and microbial pathogens in organic products with data taken from 39 studies in 13 countries worldwide.  Figure 3 lists the reported outbreaks caused by organic food in Europe and the United States in the scientific literature.

### 3.1. *Campylobacter*, *Salmonella*, and *E. coli* in Organic Products

*Campylobacter* was responsible for at least two foodborne outbreaks associated with organic raw milk and cream in 2010 and 2012 in Minnesota and California, USA, respectively. These outbreaks resulted in 7–10 illnesses, but fortunately, none of those affected died or had to be hospitalized [32]. A study of organic turkey meat in Germany showed a higher prevalence of *Campylobacter* in organic turkey meat (32.7%) than in conventional meat (19.4%) [4]. Similarly, in the United States, a *Campylobacter* prevalence of 96% was found in carcasses of chicken reared without antibiotics [33]. Furthermore, isolates fully susceptible to several antimicrobials were more frequently found in organic practice [4]. In organic production, the bacterium was also sporadically detected in eggshells [34], broiler carcasses [14], chilled retail chickens, and retail poultry samples [35, 36]. Most of the cases were associated with *C. jejuni*, while only turkey and chicken meat samples were found contaminated with *C. coli*. *C. jejuni* is considered a major cause of gastrointestinal illness worldwide and is often linked to the consumption of contaminated poultry and secondarily to some vegetables such as lettuce, spinach, green parsley or green onions, and others [37]. This contamination of fresh produce could be due to agricultural reuse of treated wastewater [38] or by cross-contamination associated with consumer handling practices [39]. In the United States, Mollenkopf et al. [22] investigated the presence of *Campylobacter* spp. in 231 retail packages of fresh boneless chicken breast. The bacterium was isolated from 12 out of 95 conventional reared samples (12.6%), 11 out of 96 (11.5%) antibiotic-free production samples, and from two out of 40 (5%) organic samples. In relation to *Salmonella*, Gambino-Shirley et al. [40] reported a multistate outbreak with a novel strain of *Salmonella* Virchow, which was linked to human consumption of a raw organic powdered smoothie product consumed as a meal replacement. The outbreak affected 24 states in North America, with 35 human cases and six confirmed hospitalizations. The main product implicated was a drinkable/meal replacement shake with 40 raw organic ingredients that have to be rehydrated before consumption. Different *Salmonella* Serovars were detected in a sample of organic moringa leaf powder and in a sample of spinach powder. In another study, Cui et al. [35] detected *Salmonella* in 61% of organic chickens, with the presence of different serovars, including Kentucky (59%), Heidelberg (33%), and Typhimurium (17%). In this case, there were no associated human cases. Likewise, *Salmonella* spp. was detected in chicken meat with a prevalence of 17.5% (7/40) in organic samples, much lower than in conventional production systems (25.2%; 24/95) [22]. In organic chicken carcasses, *Salmonella* was isolated in 13 out of 53 samples tested (25%), while in free-range chickens, the prevalence of the bacterium was higher (31%; 42/135) [41]. In another study in the United States, a *Salmonella* prevalence of 25% was found in carcasses from chickens reared without antibiotics [33]. Feed samples from organic broiler farms were also been found to be contaminated with *Salmonella*, with a prevalence of 5% (3/60) in the United States. Furthermore, a higher prevalence of multi-resistant *Salmonella* isolates was found in conventional broiler farms than in isolates from certified organic farms [42]. Recently, Horlbog et al. [43] reported the contamination with *Salmonella* serovar Jerusalem in Switzerland and Italy, with organic poultry feed being the source of the contamination of the flock. However, and as in the previous study, there were no associated human cases. In a year-long study in Louisiana, *Salmonella* serovars in Kentucky and Hadar were detected in organic chicken samples obtained from retail stores, but no human foodborne infection associated with their presence was detected [44]. Some of the isolates showed resistance to different antimicrobials, including ampicillin, ceftiofur, cefoxitin, or kanamycin, among others. In the previous study by Harvey et al. [32], a total of eight outbreaks caused by organic foods in the United States were also attributed to *Salmonella*. *Salmonella* Enteritidis was associated with two human outbreaks following consumption of organic eggs, with a total of 31 affected and eight hospitalizations in different states. *Salmonella* Typhimurium was responsible for an outbreak in Minnesota associated with vegetable soup, resulting in a total of 50 people affected, six of whom had to be hospitalized. Another outbreak caused by nut butter was associated with *Salmonella* Braenderup, resulting in a total of six poisonings. Other serovars, such as *Salmonella* Newport, Hartford, and Orangeburg, were reported to have caused a multistate outbreak linked to the consumption of organic chia seed powder. The largest *Salmonella* outbreak detected in the United States between 1992 and 2014 was associated with the serovar I 4,5,12:i-, and caused 140 cases of illness and 31 hospitalizations. The origin was organic alfalfa sprouts contaminated with the bacterium. Other cases of *Salmonella* spp. outbreaks in the states of Florida and Michigan were related with the consumption of sweet potatoes and grape tomatoes, respectively [32]. In Canada, the bacterium was detected in only one sample of organic leaf lettuce (0.9%) [27]. Another study in Minnesota reported the presence of *Salmonella* in organic lettuce and organic green paper, but also with a low prevalence (only 0.4%) [28]. In a study investigating the microbiological load of organic vegetables sold in Malaysian retail markets, *Salmonella* spp. was found in organic calamondins, carrots, and cucumbers, all with a prevalence of 7.7% (1/13 samples tested in each category). *S*. Enteritidis was only found in organic carrots with a prevalence of 14.3% (1/7 samples analyzed) [45].

Toxigenic *E. coli* is an important foodborne pathogen that has emerged in the last two decades, and fresh leafy green vegetables have been associated with the presence of serotypes such as O157:H7 [46]. In organic vegetables, the bacterium has been detected in leafy greens sold in local open-air markets and large supermarkets in Alexandria, Egypt, where organic cabbage and parsley were contaminated with *E. coli* O157:H7 with a prevalence of 16.7% [47]. Another study in the United States detected *E. coli* in a wide variety of organic products, such as tomato, lettuce, cucumber, and others. The overall reported prevalence was 9.7% (46/476), but it was shown that the prevalence of *E. coli* in certified organic produce was not statistically different from that in conventional produce [28]. In Canada, the prevalence of *E. coli* was higher in organic leaf lettuce (11.6%) than in conventional leaf lettuce (6.5%); however, no statistical difference was found between the prevalence of the pathogen in organic lettuce compared to the other products investigated [27]. In a study conducted in Spain, *E. coli* was isolated from 37.5% of organically produced eggs, out of a total of 16 samples analyzed, and only from the surface of the eggshell, but the bacterium was not found in the egg contents. Of these isolates, the highest antibiotic resistance was observed for amoxicillin-clavulanate [48]. In France, an outbreak of Shiga-toxin-producing *Escherichia coli* O104:H4 affecting eight patients with hemolytic uremic syndrome and bloody diarrhea was attributed to the consumption of organic fenugreek sprouts [49]. Similar to *Salmonella* spp., several outbreaks caused by organic foods have been associated with *E. coli* O157:H7 in the United States. The foods involved included vegetables such as lettuce or spinach and raw milk and cream, and this resulted in a total of 299 illnesses and 133 hospitalizations [32]. In Denmark, an organic fermented meat sausage was the source of non-O157 Shiga toxin-producing *E. coli* outbreak in 20 children [50].

### 3.2. Listeria, Staphylococcus, and Enterococcus in Organic Products

An outbreak of listeriosis has been reported in Pavia, Italy, affecting a total of six patients, one of them fatal. Some of these patients had recently consumed organic or homemade cheese, and a subsequent epidemiological investigation identified an organic cheese production farm as a possible source of the outbreak. The results of a retrospective whole genome sequencing study of *Listeria monocytogenes* (*L. monocytogenes*) raised the hypothesis that almost two patients were infected after consuming cheese produced on this organic farm [51]. Another study investigating the prevalence of *L. monocytogenes* on organic and conventional farms found a bacterial prevalence in cut pork of 3% and 4%, respectively. However, when other types of samples, such as feed and litter, rectal swabs, intestinal contents, or carcasses, were analyzed, the prevalence was found to be higher in organic than in conventional pig production, both on the farm and at the slaughterhouse [26]. In a study in northern Spain, samples of organic poultry meat collected in supermarkets and butcher shops were found to be contaminated with *L. monocytogenes*, with a prevalence of 49.1% (27/55 samples analyzed). However, in this case, the authors found no difference between the prevalence of this bacterium in organic poultry meat compared to conventional poultry meat [23]. In Norway, from 179 samples of organically grown lettuce, *L. monocytogenes* serogroups 1 and 4 were isolated from a total of two samples (1.1%) [52]. In Asia, Kuan et al. [45] found a prevalence of *L. monocytogenes* in organic vegetables of 2.7% (two positive samples out of 75, one positive organic cabbage and one positive organic white radish), while the prevalence of *Listeria* spp. was slightly higher (6.7%, five out of 75 samples), one positive organic cabbage, two positive organic lettuce, and two positive organic white radish samples). Similarly, in Korea, Tango et al. [53] described a prevalence of *L. monocytogenes* in four out of 63 samples (6.4%) in organic romaine lettuce and spinach, with only slight differences between organic and conventional products.

Isolation of methicillin-resistant *Staphylococcus aureus* (*S. aureus*) in bulk milk tanks from organic dairy herds in Germany was described with a prevalence of 1.7%. This prevalence was lower than that found in conventional production (9.7%), while the authors also observed an effect of herd size and region on the presence of the bacterium [29]. Also, in Iran, the presence of *S. aureus* was described with a prevalence of 27% in milk and cheese samples collected from farms and milk collection points. SEA, which is the most common staphylococcal enterotoxin associated with food poisoning, was found in 12.9% of the recovered isolates, suggesting a potential risk to human health [54]. An observational study to compare the prevalence of *S. aureus* in bulk tank milk from organic farms in Wisconsin and Denmark reported a prevalence of the bacterium of 64.4% and 50%, respectively. In the same study, significant differences were detected between organic and conventional production, specifically for ciprofloxacin in Wisconsin and avilamycin in Denmark [55]. In Greece, a study investigating the presence of different pathogens, including *S. aureus*, in milk from sheep and goat farms found a prevalence of this bacterium of 76% (19/25) in organic milk samples. Although antibiotic resistance detected in this study was low, a higher percentage was observed among strains from conventional compared to organic farms [56]. Other studies on *S. aureus* in meat found no difference in the recovery of the bacteria in organic and conventional poultry samples. In organic production, the bacterium was detected in 67.3% of the samples, but 32.7% of these positive samples were below the detection limit [23]. Regarding organic vegetables, *S. aureus* was reported in lettuce (6.34%), spinach (6.34%), and sesame leaves (7.93%) [53]. *Enterococcus* spp. (including *Enterococcus faecalis*) are often investigated to determine antibiotic resistance profiles and spread in farm animals. In Korea, Kim et al. [24] reported that *Enterococcus* spp. contamination rates, as well as the level of multidrug resistance isolates, were lower in organic chicken carcasses than in those from conventional production. Similarly, in Spain, Miranda et al. [21] found lower rates of antimicrobial resistance in *Enterococcus* spp. isolated from organically produced chicken and turkey meat than in conventionally reared animals. In contrast, in the United States (Tennessee), another study reported that the prevalence of *Enterococcus* spp. in organic chicken was almost twice as high (62.5%) as in conventional chicken (37.5%). However, the number of antibiotic resistance isolates was lower in organic chicken (31%) than in conventional chicken (43.6%) [25]. Schwaiger et al. [30] investigated the prevalence and antimicrobial resistance patterns of *Enterococcus* in organic and cage poultry egg samples in Germany. The results showed a higher prevalence of the bacteria in egg contents from conventional layer flocks (27.5%; 11/40) compared to organic production (20%; 8/40). In eggshells, the same prevalence (60%; 24/40) was found in both types of systems. In addition, multiple antibiotic resistances were statistically more frequent in *E. faecalis* from conventional production.

### 3.3. Other Pathogens in Organic Products

In the United States, an outbreak of *Clostridium botulinum* (*C. botulinum*) was previously reported in several states associated with pasteurized carrot juice, with a total of four affected persons requiring hospitalization, one of whom died [32]. In fresh produce without chemical additives in Egypt, hepatitis A virus was detected in strawberries and green leafy vegetables with a prevalence of 48% and 31.2%, respectively. The same study also investigated the presence of norovirus genogroups I and II in these samples, finding a prevalence ranging from 20% to 40% [57].

## 4. RASFF for Food and Safety Alerts

**Figure 4:**
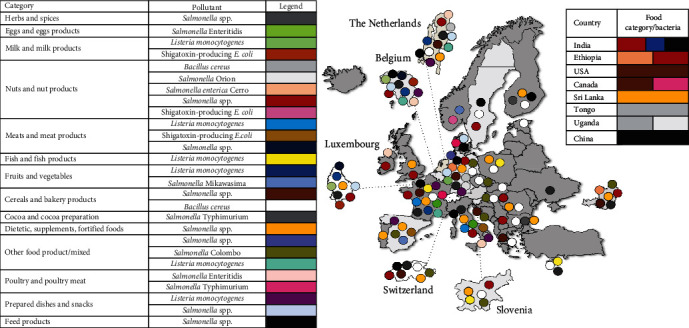
RASFF notifications in organic food and feed by country (including the country of origin, notification, and/or distribution), and by the type of pathogen.

Figure 4 compiles RASFF alerts in organic food and feed by country (origin, notification, and/or distribution), and type of pathogen.

Regarding organic food product category, a total of 13 groups were identified: (1) cereals and bakery products; (2) cocoa and cocoa preparations, coffee and tea; (3) dietetic foods, food supplements, and fortified foods; (4) eggs and eggs products; (5) fish and fish products; (6) fruits and vegetables; (7) herbs and spices; (8) milk and milk products; (9) meat and meat products (other than poultry); (10) nuts, nut products, and seeds; (11) other food product/mixed; (12) poultry meat and poultry meat products; (13) prepared dishes and snacks. In organic feed products, a total of three groups were identified: (1) organic soybean, (2) organic rape, and (3) L-isoleucine 3c383 additive.

### 4.1. RASFF Alerts in Organic/Bio Food

A total of 42 food alerts were reported in organic/bio food were notified in the period under study, and 37 of these (88.1%) were classified as serious risk decisions (Table 2). In 42.9% of these cases, the basis was the company's own detection, and they were classified as alert notifications. Among the measures taken, public warning was carried out in 16.6% of the notifications, followed by official detention of the product in 14.3% of the cases. Other actions taken more frequently were retention of the product by the operator and withdrawal from the market, both in 11.9% of all notifications. Germany was the country that made the most notifications in this category (40.5%), followed by Belgium (14.3%), France (9.5%), Slovenia (9.5%), and the Netherlands (9.5%). Regarding the origin of the product concerned, India was the country with the highest number of products involved (14.3%), followed by Belgium (9.5%), Germany, Spain, France, Italy, and Ethiopia (7.1%), in equal percentage. Nuts, nut products, and seeds were the main products concerned (11 out of 42 notifications, 26.2%), followed by dietetic foods, food supplements, and fortified foods (five out of 42 notifications, 11.9%), and meat and meat products other than poultry (five out of 42 notifications, 9.5%). *Salmonella* was the predominant pathogen reported in organic/bio food, with a total of 29 notifications (69%) and, within this genus, *Salmonella* spp. (50%), *S*. Typhimurium (4.8%), *S*. Enteritidis (4.8%), *S*. Mikawasima (2.4%), *S*. Colombo (2.4%), and *S*. Cerro (2.4%) were reported in 11 product categories (Table 2). The second most frequently detected pathogen was *L. monocytogenes*, with eight out of 42 (19%) notifications in six product categories, including fish (smoked salmon), vegetables (beetroots and salad leaves), dairy products (cheese), meat products (deli meats), and other foods (ready-to-eat preparations) and other prepared dishes (vegan cheese). Shiga toxin-producing *E. coli* was reported with a prevalence of 7.1% (three out of 42 notifications), and the foodstuffs involved were raw milk, goat cheese, beef, and leek seeds for sprouting. Finally, the last pathogen implicated in organic food notifications was *B. cereus*, which was found in bakery products and sesame seeds with an overall prevalence of 4.8%. In the category of eggs and eggs products, we found the only notification of food poisoning detected in organic foods, with 31 human cases and due to the consumption of organic eggs contaminated with *S*. Enteritidis. According to the information collected at the RASFF window, the eggs were suspected to have originated from Italy and distributed in France, which also reported the outbreak. So far, no *S*. Enteritidis from this cluster had been isolated in France.

### 4.2. RASFF Notifications in Organic/Bio Feed

When analyzing feed materials of organic/bio origin, a total of 26 notifications were identified, but all were classified as non-serious risk, except for one notification listed as undecided and one notification classified as the presence of antibiotic resistance genes in a feed additive, which was found to have no associated risk (Table 3). For most of these notifications (92.3%), the basis was the company's own detection, and they were classified as information/notification for follow-up. For 42.3% of the cases, the measures taken were physical/chemical treatment, including acid treatment of the feed, while in 26.9% of the notifications, the feed product was detained by the operator. The top reporting countries in this category were Germany (53.8%), followed by Finland (19.2%) and Sweden (15.4%). In relation to the origin of the products concerned, Italy was the country with the highest number of products involved (23.1%), followed by the Netherlands and China, both of them with the same percentage (15.4%). Only two categories of products were implicated with the presence of microbial pathogens, including organic soybean (18 out of 26 notifications, 69.2%) and organic rape (eight out of 26 notifications, 30.8%). In organic/bio feed, the only microbial pathogen detected was the genus *Salmonella*. *Salmonella* spp. was reported in 14 out of 26 notifications (53.8%), while *S. enterica* was reported in 12 out of 26 notifications (46.2%) with 12 different serovars identified. Only in one feed additive (L-isoleucine 3c383), the notification was associated with the presence of antibiotic-resistance genes. The product originated from China, was distributed and notified by Belgium, and was withheld by Norway, although finally, no risk was associated with this notification.

## 5. Discussion and Concluding Remarks

The growing popularity of organic food production in response to consumer demand is a promising trend driven by the perception of safer and higher-quality products. However, the scientific evidence supporting the food safety benefits of organic products is still inconclusive. Although organic farming standards promote improved antimicrobial stewardship in livestock, restricting the use of antibiotics to only when necessary, the precise impact on food safety remains an area of ongoing study. First, in this review, the main microbial hazards associated exclusively with organic products were analyzed as reported in the literature. Second, using the EU Rapid Alert System, we identified the main hazards found in organic feed and food, and we summarized the notifications in relation to these products over the last 3 years in Europe.

A significant finding of the studies reviewed in this work is the decreasing trend of multidrug-resistant strains in favor of organic production compared to conventional methods. This is encouraging, as it suggests that organic practices may play a role in reducing the circulation of antimicrobial resistance strains, which is crucial in the context of the expected global increase in antimicrobial consumption. Limiting the development and spread of antimicrobial-resistant pathogens is of vital importance to safeguard public health and maintain the effectiveness of existing antimicrobial treatments.

On the other hand, regarding the presence of microbial pathogens in organic food products, the results of different studies present contradictory results. This inconsistency makes it difficult to draw definitive conclusions on the impact of organic production on the prevalence of microbial contaminants in the final product. Therefore, further robust research in this area is, therefore, essential to obtain a comprehensive understanding of the relationship between organic practices and microbial contamination by pathogens. EU organic farming standards provide a comprehensive framework for regulating agricultural products, including aquaculture and yeast, at all stages of the production process. Despite the strict regulations, RASFF notifications reveal that *Salmonella* was the predominant pathogen reported in both organic food and feed, with seeds being the most affected product. *Salmonella* is a major cause of foodborne illness worldwide and poses a considerable risk to public health. The detection of a single foodborne outbreak associated with *Salmonella* Enteritidis in eggs during the study period suggests that current safety measures may be effective in controlling and preventing large-scale outbreaks. However, continued surveillance and targeted interventions are necessary to further reduce the risk of foodborne diseases. In addition, a significant proportion of the food products with alerts originated from India, highlighting the importance of ongoing surveillance and control measures in light of the growing international trade in organic products. The fact that seeds are the most affected food product and that a considerable percentage of food products with descriptions originate from third countries points to specific areas requiring attention and further research.

To address the complexities of food safety in organic production, sanitary control of organic food products remains vital. Understanding how organic practices influence the occurrence of microbial contamination, the emergence of certain bacteria, and the spread and the spread of antibiotic-resistant genes will guide the development of targeted and effective food strategies. In addition, to gain a comprehensive understanding of the impact of organic food production on microbial contamination, researchers must take into account factors such as farm management practices, soil health, climate, and regional differences. Comparative studies between organic farms in diverse geographical locations and conventional farms can provide valuable insights into the complex interactions between agricultural practices and microbial ecology.

One significant limitation of this study is its heavy reliance on scientific literature as a primary source of information on reported outbreaks. Ideally, access to country- or community-specific public health databases would provide more comprehensive data. However, this approach may pose some problems, particularly due to language barriers, and may only be feasible for a limited number of countries, such as the European Union, to maintain consistency with the use of RASFF. In addition, it is important to recognize that our study's focus on the RASSF system and European regulatory standards may limit the generalizability of our findings to regions with different food safety and contaminant criteria, although we include relevant studies from other countries to provide a broader perspective. Another limitation of this work is that not all outbreaks are dated due to the inconsistent availability of this information from the consulted sources.

In conclusion, this study provides valuable information about the potential risks and challenges associated with pathogenic microorganisms in organic food production in recent years. The results highlight the need for continuous monitoring and improvement of food safety measures in organic systems to ensure that organic products meet high safety standards. As the world moves toward a greener and more sustainable economy, it is crucial to understand and address food safety issues in organic production. Emphasizing the responsible use of antimicrobials, implementing strict hygiene practices, and improving traceability mechanisms are essential steps to mitigate the risks associated with pathogenic microorganisms in organic products. Integrating responsible antimicrobial stewardship and effective monitoring tools can help mitigate foodborne pathogens' risks and safeguard public health. As organic food production and international trade continue to grow, fostering a holistic and science-driven approach will be crucial in harnessing its potential for a sustainable and safe food future.

## Figures and Tables

**Table 1:** Comparison of the presence of bacteria and antibiotic resistance genes between conventional and organic foodstuffs.

Product category	Organic/bio product	Bacteria	Prevalence in organic products compared to conventional products	Antibiotic resistance in organic products compared to conventional products	Reference
Meats	Turkey meat	*C. jejuni*	Higher prevalence in organic samples	Lower antibiotic resistance rates in organic samples	Tenhagen et al. [4]
		*Enterococcus* sp.	No data available	Lower antibiotic resistance rates in organic samples	[21]
	Chicken products	*E. coli*	No data available	No statistical differences in antibiotic resistances	[7]
		*Campylobacter* spp.	Lower prevalence in organic samples		[22]
		*Salmonella* spp.	Lower prevalence in organic samples		
		*L. monocytogenes*	No statistical differences in the prevalence		[23]
		*Enterococcus* sp.	Lower prevalence in organic samples	Lower antibiotic resistances in organic samples	[24]
			Higher prevalence in organic samples		[25]
				Lower antibiotic resistance rates in organic samples	
				Lower antibiotic resistance rates in organic samples	[21]
	Pork products	*L. monocytogenes*	Lower prevalence in organic samples		[26]
	Chicken breast	*Salmonella*		Lower antibiotic resistance rates in organic samples	[5]
	Ground beef ground turkey	*Campylobacter, Enterococcus*			
	Pork chops	*E. coli*			

Vegetables	Lettuce	Tetracycline and sulfadiazine-resistant bacteria		No statistical differences in antibiotic resistances	[8]
		*E. coli*	Higher prevalence in organic samples		[27]
	Variety of vegetables	*E. coli*	No statistical differences in the prevalence		[28]

Milk and milk products	Fresh cheese	*Staphylococcus* spp.		No statistical differences in antibiotic resistances	[26]
	Milk	*S. aureus*	Lower prevalence in organic samples		[29]

Eggs	Egg content	*Enterococcus* spp.	Lower prevalence in organic samples		Schwaiger et al. [30]
	Eggshell		No statistical differences in the prevalence		
	Eggshell and egg content	*E. faecalis*		Lower antibiotic resistance rates in organic samples	

**Table 2:** EU RASFF notifications in organic/bio food products between 2020 and 2022.

Product category	Pathogen	Organic/bio product	Country			Type	Measures (human cases)	Risk decision	Year and Ref.
			Notifying	Origin	Distribution				
Cereals and bakery products	*Salmonella* spp.	Red berries protein bars	CH	CA	FR, CH	3C	NSL	Undecided	2022.2746
		Organic tiger nut flour (porridge)	DE	NE	>3 countries	3B	PW	Serious	2021.1976
	*Bacillus cereus*	Barley grass powder	DE	HU	>3 countries	4C	MR	Not serious	2021.6580

Cocoa and cocoa preparations, coffee, and tea	*Salmonella* Typhimurium	Organic linden flower infusion	FI	BG	–	4C	WM	Not serious	2021.0827

Dietetic foods, food supplements, and fortified foods	*Salmonella* spp.	Moringa powder	DE	LK	>3 countries	3B	DO	Serious	2022.2231
		Supplement ashwagandha	SI	SI	–	4B	WM	Serious	2021.6277
			SI	IN	–	4B	OD	Serious	2021.5238
			DE	IN	DE	3D	WR	Serious	2021.0891
		Shatavari powder	DE	IN	>3 countries	3B	IR	Serious	2021.2600

Eggs and eggs products	*Salmonella* Enteritidis	Eggs	FR	IT	FR	1B	NM (31)	Serious	2020.0125

Fish and fish products	*L. monocytogenes*	Frozen smoked salmon	CY	PL	CY	4D	NM	Serious	2020.1049
		Smoked salmon	FR	UN	FR, SI	3B	RC	Serious	2020.1631

Fruits and vegetables	*L. monocytogenes* beetroots	Chilled cooked	NL	NL	BE	3B	PW	Serious	2021.2328
		Salad leaves	BE	FR	BE, LU	3D	IA	Serious	2020.2682
	*Salmonella* Mikawasima	Little gem lettuce	NO	ES	NO	3D	RC	Serious	2020.0765

Herbs and spices	*Salmonella* spp.	Salt and pepper mix	DE	DE	CZ	3B	NM	Serious	2022.2690
		Coriander seeds	DE	UN	DE	3B	NSL	Serious	2022.2397
		Horsetail	DE	DE, IT, UA	>3 countries	3B	RC, WM	Serious	2021.5945

Milk and milk products	*L. monocytogenes*	Emmental cheese	BE	DE	BE	3B	IR	Serious	2022.1939
	STEC	Raw milk goat's cheese	BE	BE	LU	4B	PW	Serious	2020.1072

Meat and meat products (other than poultry)	STEC	Bovine meat	IT	ES	IT	4B	D	Serious	2021.2303
	*Salmonella* spp.	Chilled pork meat	BE	BE	BE, LU	3B	PW	Serious	2021.4316
	*L. monocytogenes*	Mortadella and cooked jam	FR	IT	–	3B	PW	Serious	2020.2321
	*Salmonella* spp.	Minced pork and bio chipolata	BE	BE	BE, LU	3B	PW	Serious	2020.5896

Nuts, nut products, and seeds	*Salmonella* spp.	Nettle seeds	DE	KO	>3 countries	3B	MR	Serious	2022.0407
	*Bacillus cereus*	Sesame seeds unhulled	DE	TG	NL	3B	DO	Serious	2022.0133
	*Salmonella* spp.	Flaxseed	FR	NL	FR	3D	IA	Serious	2022.1580
	*Salmonella* Orion	Sesame seeds	DE	UG	DE	2A	OD	Serious	2022.6017
	*Salmonella* spp.	Sesame seeds	NL	UG	–	2A	OD	Serious	2021.6740
	*Salmonella enterica* cerro	Sesame seeds	DE	ET	DE	2A	OD	Serious	2021.5397
	*Salmonella* spp.	Sesame seeds	DE	ET	DE	2A	PT	Serious	2020.5495
			SI	IN	AT	2A	OD	Serious	2020.3260
			SI	IN	SI	2A	NM	Not serious	2020.3090
			NL	ET	–	2A	OD	Serious	2020.1735
	STEC	Leek seeds for sprouting	NO	CN	NO	3C	DO	Undecided	2020.0500

Other food products/mixed	*Salmonella* Colombo	Tiger nut flour	DE	ES	>3 countries	4B	PW	Serious	2021.5807
	*Salmonella* spp.	Bacopa monnieri powder	DE	IN	DE	3D	DO	Serious	2021.4870
	*L. monocytogenes*	Ready-to-eat preparations	BE	BE	FR, NL	3B	WM	Serious	2021.2496

Poultry meat and poultry meat products	*Salmonella* Enteritidis	Chicken thigh fillets	NL	IT	BE, NL, IE	3B	WR	Serious	2022.0960
	*Salmonella* Typhimurium	Whole chicken	DK	FR	DK	3D	NM	Serious	2020.4674

Prepared dishes and snacks	*Salmonella* spp.	Cheese-flavored protein chips	DE	DK	>3 countries	3B	DO, RC, IR, PW	Serious	2022.5519
	*L. monocytogenes*	Vegan cheese alternative	DE	FR	>3 countries	3B	WM, RC, D	Serious	2022.2311

STEC: Shigatoxin-producing *Escherichia coli*. AT (Austria); BE (Belgium); BG (Bulgaria); CA (Canada); CH (Switzerland); CN (China); CY (Cyprus); CZ (Czech Republic); DK (Denmark); DE (Germany); ET (Ethiopia); ES (Spain); FI (Finland); FR (France); HU (Hungary); IE (Ireland); IN (India); IT (Italy); KO (Kosovo); LK (Sri Lanka); LU (Luxembourg); NE (Niger); NL (Netherlands); NO (Norway); PL (Poland); SI (Slovenia); TG (Togo); UA (Ukrania); UG (Uganda), UN (Unknown). 1. Food poisoning notification. 2. Food (border control-consignment detained). 3. Company's own check. 4. Official control on the market. 5. Consumer complaint. A. Border rejection notification. B. Alert notification. C. Information/notification for follow-up. D. Information/notification for attention. D, destruction; DO, detained by operator; IA, informed authorities; IR, informed recipients; MR, monitoring of the recall/withdrawal; OD, official detention; PT, physical/chemical treatment, acid treatment, or heat treatment; PW, public warning; RC, recall from consumer; NM, no measures found for this notification; NSL, no stock left; WM, withdrawal from the market; WR, withdrawal from the recipients.

**Table 3:** RASFF notifications in organic/bio feed products between 2020 and 2022.

Organic/bio product	Pathogen	Country			Type	Measures (human cases)	Risk decision	Year. Ref
		Notifying	Origin	Distribution				
Organic soybean	*Salmonella* spp.	SE	–	–	3D	PT	Not serious	2022.6072
		DE	CN	DE, NL	3C	PT	Not serious	2022.4445
		SE	NL	SE, DK	3C	PT	Not serious	2022.3207
		DE	IT	DE, BE	3C	IA	Not serious	2022.0130
		DE	IT	BE, FR, DE, SE	3C	IR	Not serious	2021.6386
		DE	BE	BE	3C	DO	Not serious	2020.5502
		DE	NL	DE	3C	NM	Not serious	2020.5321
		FI	IN	FI	2A	REC	Not serious	2020.0800
		DE	IT	DE	3C	DO	Not serious	2020.0710
		DE	CH	DE, SE	3C	DO	Not serious	2020.0708
		SE	NL	SE	3C	PT	Not serious	2020.2149
	*S. enterica* serovar Agona	SE	CN	NL	3C	PT	Not serious	2022.3992
		BE	NL	BE	3C	PT	Not serious	2022.3238
	*S. enterica* serovar Senftenberg *S. enterica* serovar Mbandaka	DE	CN	DE, NL	3C	IA, PT	Not serious	2022.4233
	*S. enterica* serovar Abaetetuba	DE	CN	BE, NL	3C	IA, PT	Not serious	2022.1374
	*S. enterica* serovar London	DE	IT	DE, FI	3C	PT	Not serious	2022.0118
	*S. enterica* serovar Korlebu	DE	IN	DE, FR, BE	3C	IA	Not serious	2021.4725
	*S. enterica* serovar Tennessee	DE	CH	DE, NL	3C	DO	Undecided	2020.1054

Organic rape	*S. enterica* serovar Jerusalem *S. enterica* serovar Kedougou	FI	IT	DK	3C	PT	Not serious	2022.1857
	*S. enterica* serovar Senftenberg	DE	DE	DK	3C	DO	Not serious	2022.1487
	*S. enterica* serovar Bradford	CH	DE	CH	3C	NM	Not serious	2022.0873
	*Salmonella* spp.	DE	IT	DE	3C	DO	Not serious	2021.5546
		FI	BE	FI	3C	PT	Not serious	2021.1761
		FI	FR	FI	3C	PT	Not serious	2020.0401
	*S. enterica* serovar Anatum	FI	FR	FI	3C	PT	Not serious	2021.2498

L-isoleucine 3c383 additive	Antibiotic resistance genes	BE	CH	BE	3C	DO	No risk	2021.3378

BE (Belgium); CH (Switzerland); CN (China); DK (Denmark); DE (Germany); FI (Finland); FR (France); IN (India); IT (Italy); NL (Netherlands); SE (Sweden). 1. Food poisoning notification. 2. Food (border control-consignment detained). 3. Company's own check. 4. Official control on the market. 5. Consumer complaint. A. Border rejection notification. B. Alert notification. C. Information/notification for follow-up. D. Information/notification for attention. DO, detained by operator; IR, informed recipients; IA, informing authorities; NM, no measures found for this notification; PT, physical/chemical treatment, acid treatment.

## Data Availability

No underlying data were collected or produced in this study.
